# Health beliefs and attitudes toward Influenza and COVID-19 vaccination in Portugal: a study using a mixed-method approach

**DOI:** 10.3389/fpubh.2023.1331136

**Published:** 2024-01-19

**Authors:** Ana João Santos, Irina Kislaya, Carlos Matias-Dias, Ausenda Machado

**Affiliations:** ^1^CINTESIS@RISE, Department of Education and Psychology, University of Aveiro, Aveiro, Portugal; ^2^Department of Infectious Diseases Epidemiology, Bernhard Nocht Institute for Tropical Medicine (BNITM), Hamburg, Germany; ^3^Epidemiology Department, National Institute of Health Doctor Ricardo Jorge, Lisboa, Portugal; ^4^Comprehensive Health Research Center (CHRC), NOVA University of Lisbon, Lisbon, Portugal; ^5^Public Health Research Centre, National School of Public Health, NOVA University of Lisbon, Lisbon, Portugal

**Keywords:** influenza vaccine, COVID-19 vaccine, Health Belief Model, vaccine uptake, mixed-methods

## Abstract

**Introduction:**

Vaccination is one of the most effective population strategies to prevent infectious diseases and mitigate pandemics, and it is important to understand vaccine uptake determinants since vaccine hesitancy has been increasing for the past few decades. The Health Belief Model (HBM) has been widely used for understanding vaccination behavior. The current study aimed to assess influenza vaccine (IV) non-uptake and attitudes toward COVID-19 vaccination, two important respiratory diseases with similar symptoms, and routes of transmission in the Portuguese population.

**Methods:**

We conducted a cross-sectional study using a panel sample of randomly chosen Portuguese households. A total of 1,050 individuals aged 18 years and over responded to a telephone or online questionnaire. Through a mixed-method approach, we employed thematic content analysis to describe reasons for not taking the IV, considering the HBM dimensions, and quantitative statistical analysis to estimate IV and COVID-19 vaccine coverage.

**Results:**

The IV uptake for the overall population was 30.7% (CI 95%: 26.5, 35.2). Susceptibility was found to be a main factor for IV non-uptake, followed by barriers, such as stock availability and fear of adverse effects. The uptake of the COVID-19 vaccine was very high in the study population (83.1%, CI 95%: 13.6%−20.9%). There was a high perception of COVID-19-associated severity and fear of the consequences. Individuals who reported IV uptake seemed to perceive a higher severity of COVID-19 and a higher benefit of taking the COVID-19 vaccine for severe complications.

**Discussion:**

Thus, the population does not seem to consider influenza to be a health risk, as opposed to COVID-19, which is considered to be a possibly severe disease. The association between IV uptake and COVID-19 perceptions highlights that an overall attitude toward vaccination in general may be an important individual determinant.

## 1 Introduction

Vaccination has long been regarded as one of the most effective public health approaches to prevent infectious diseases and restrain pandemics (1), either by reducing disease incidence or the complications and deaths caused by infectious agents (2). The success of this measure relies on both vaccine effectiveness and population-wide uptake (3).

Vaccination uptake has been, particularly for the last couple of decades, subject to increasing attention from scientific and public health communities due to vaccine hesitancy (4, 5). Vaccine hesitancy refers to the delay in acceptance or refusal of vaccines despite the availability of vaccination services (6).

The problem of vaccine hesitancy is not new, as the presence of opposing movements in the history of the vaccine against smallpox, measles, rubella, and, more recently, the influenza vaccine (IV) and the vaccine against COVID-19 have been identified (4, 7, 8). Since vaccines appeared in medical practice at the end of the 18th century, we have witnessed the appearance of controversies that arise intermittently in different parts of the world, with arguments based on theology, beliefs, and skepticism, among others (7, 8).

During the COVID-19 pandemic, there was increased awareness of the vaccine development process and of the importance of vaccines for controlling infectious diseases. This awareness may impact not only the COVID-19 vaccine uptake but also the uptake of other vaccines, such as the seasonal influenza vaccine (9).

Several studies have focused both on IV and COVID-19 vaccine uptake, including hesitancy, acceptance, or refusal (9–13). Seasonal influenza and COVID-19 are infectious respiratory diseases with similar symptoms (10) and routes of transmission (14, 15). In addition, the high-risk populations for both diseases include similar groups, namely, older adults and people with chronic conditions (10).

Since 2001/2002, in Portugal, an at-risk-based influenza vaccination program has been implemented (16). The National Directorate for Health clinical guidelines recommends influenza vaccination to individuals aged 65 and over and those at higher risk for influenza complications (chronic and immunocompromised patients older than 6 months of age and pregnant women), to whom the vaccine is offered free of charge (17). The seasonal influenza vaccination campaigns start every year in early October and develop throughout the winter (16).

For the COVID-19 vaccination campaign that started at the end of December 2020, the target population for the first and second vaccination phases included people over 50 years of age with heart failure, coronary heart disease, kidney failure, or respiratory disease, people over 65 (with or without pathologies), and people aged between 50 and 64 with diabetes, active malignant neoplasia, chronic kidney disease, liver failure, obesity, or hypertension (18). The campaign's third phase included the entire Portuguese population above 6 years of age.

Considering the potential extra pressure on healthcare services, several specialists alerted, during the pandemic, to the importance of avoiding the emergence of coinfection of influenza and COVID-19 (9, 13, 14).

Influenza vaccine coverage varies globally, and for the most part, it remains below the target proposed by the WHO (19). Similarly, several COVID-19 vaccine studies indicated hesitant populations, with individuals unwilling or unsure to take the vaccine (20–22). A systematic review of 16 studies (11 conducted in Asia, three in North America, and 2 in Europe) showed an overall vaccination hesitancy rate for the COVID-19 vaccine of 33.2% (95% CI 24.7–41.4%, SD = 17.35), ranging from 8.4% to 60.6% (23).

Considering that vaccination coupled with personal protective behaviors are the most effective and cost-effective strategies for public health protection from both illnesses (10), understanding the determinants and barriers to vaccine acceptance is important (11). Vaccine uptake is not the same for all vaccines, but even for the same vaccine, it can originate from different arguments and rationales (7, 24). Hence, it is important to understand and distinguish between general or similar factors associated with vaccine uptake and the determinants related to specific vaccine types and products.

Vaccine hesitancy has a global dimension, but it includes several factors that vary in time and context, making it a complex, multifaceted, and dynamic social process that reflects multiple factors (4, 7, 23), such as structural (e.g., vaccine cost, healthcare system), contextual, cultural, and social norms and values (e.g., religious beliefs) at the group and individual level.

Because people's health beliefs are major determinants of vaccine hesitancy (23), the Health Belief Model (HBM) is one of the most widely used models for understanding vaccination behavior in relation to influenza (25–30) and COVID-19 (13, 23, 31–36). The HBM is a theoretical model that conceptualizes health-related behavior as the combination of several factors, namely, perceived susceptibility, perceived severity, perceived benefits, perceived barriers, and clues to action (37). When compared with other models that explain behavior and resulting action, the HBM has been specifically developed to focus on preventative health research (34, 37). When applied to vaccines, it describes and predicts how people evaluate the risk of susceptibility to a disease that a vaccine protects against, the severity and complications related to the disease, and the dangers associated with the vaccine uptake (38).

The HBM as a theoretical framework for the uptake of the IV and COVID-19 vaccine may help to identify similarities and differences between the two and can be helpful to design tailored strategies to target vaccine hesitancy.

The aims of the present study were to explore the attitudes and beliefs toward influenza and COVID-19 vaccination using the HBM. Specifically, it sought to assess IV non-uptake and the overall attitude toward COVID-19 vaccination in the Portuguese population in 2021 during the COVID-19 primary-series vaccination campaign.

## 2 Materials and methods

### 2.1 Study settings

The study comprised a mixed-method approach, including both qualitative and quantitative analysis.

We conducted a cross-sectional study using the panel of Portuguese families Em Casa Observamos Saúde (ECOS/At home, we observe health), a probabilistic household sample developed by the National Health Institute Doutor Ricardo Jorge (INSA) and in place since 1998/99 (39–42).

The ECOS panel is a random sample of Portuguese households stratified and homogeneously distributed by seven regions that comprise the seven Statistical Territorial Units of Portugal. The ECOS sample is based on a dual-frame design, with a random selection of landline and mobile phone numbers generated through random digit dialing.

The panel used in this study was first sampled in 2018 and refreshed in the spring of 2021, achieving a final number of 1,537 household units.

### 2.2 Data collection

Data were collected between June and August 2021 through a questionnaire applied via computer-assisted telephone interview (CATI) or computer-assisted web interview (CAWI) to one member of each household unit aged 18 or more years.

The questionnaire covered demographic information, presence of chronic conditions (asthma, allergic diseases, chronic obstructive pulmonary disease, diabetes, obesity, high blood pressure, ischemic heart disease, stroke, cancer, osteoarticular disease, depression, neurodegenerative disease, kidney disease, liver disease, spine disease, and immunosuppression [including chemotherapy, transplantation and HIV treatments)], influenza and COVID-19 vaccine uptake during 2020/21, motivations of the non-vaccinated, and items of the HBM on COVID-19 vaccination.

The items used to assess the HBM for COVID-19 vaccination were adapted from a questionnaire already validated for IV (27). It included 13 closed-ended questions describing the five dimensions of the HBM (severity, susceptibility, barriers, benefits, and cues to action). The respondents were asked of their level of agreement through a five-point Likert scale (from 1—totally disagree to 5—completely agree).

Motivations for not having been vaccinated against influenza were assessed by an open-ended question in order to not prompt the response.

### 2.3 Analysis

We used thematic content analysis to describe the qualitative data obtained through the open-ended question on reasons for not having been vaccinated. Thematic content analysis allows for the analytical examination of narrative materials by breaking texts into relatively small units of content (43). Individuals' responses were first transcribed verbatim by the interviewers, and then, the first researcher systematically coded and categorized the different thematic units. The analysis was conducted considering the HBM dimensions, but the thematic units were not defined a priori. These were developed from the analyses of the open-ended question responses and their content (e.g., a response mentioning “getting sick with the flu” as a reason not to take the vaccine originated the thematic unit “side effects”). The thematic units were developed based on the consensual themes and repeatedly assessed against the empirical material. The researcher further explored all text segments in each category with more in-depth categorical and theoretical–substantive coding categories—additional categories were developed or modified whenever necessary.

A researcher triangulation process (44) was implemented to improve the scientific validity of the coding the results. The different units (uncoded segments) were provided to a second researcher, in addition to the description of categories. This second researcher coded each of the segments provided in the identified categories. The coding was compared to the first researcher's results, and the discrepancies (mismatched segments, *n* = 28, 10.2%) were discussed by the three researchers until a consensual decision was reached. In some cases, consensus was achieved by changing the description of categories and/or their name, and in very few cases, the statement was coded differently. No software was used. The final coding tree corresponded to the consensus reached among the members of the research team. We then mapped each thematic unit to one of the five dimensions of the HBM when possible to enable comparison between these results and those of the closed-ended HBM questionnaire of COVID-19 vaccination.

The descriptive statistical analysis of all the categorical variables by frequencies and percentages and central tendency statistics for the closed-ended items of the HBM questionnaire resulted in estimates of the proportions of people vaccinated with the IV and COVID-19 vaccine, and in the case of the IV, these were stratified by sex, age group, and presence of chronic conditions.

All estimates were weighted to account for sampling design and to match the distribution of the Portuguese resident population in terms of age group and sex.

We used design-adjusted versions of the chi-square test and Student's *t*-test to evaluate the associations between participant characteristics, vaccine uptake, and HBM dimensions (45, 46). A significance level of <0.05 was considered for the employed tests. All calculations were performed using the [svy] module of the statistical program STATA/SE 15.1 for Windows^®^ (Stata Corp, College Station, TX, USA) to account for the sampling design.

### 2.4 Ethical considerations

The ECOS panel of the families' survey protocol was approved by the Portuguese Data Protection Authority (approval number 1451/2010, April 12, 2010).

## 3 Results

From the 1,537 household units that were part of the panel, we obtained 1,050 responses in the spring 2021 wave, leading to a response rate of 68%.

Overall, 53.2% (CI 95%: 48.2, 58.2) were women, about half were employed 53.7% (CI 95%: 48.3, 59.0) and the majority reported at least one chronic condition diagnosed by a health professional (82.1%; CI 95%: 10.3, 20.4; Table 1).

**Table 1:** Health beliefs and attitudes toward influenza and COVID-19 vaccination in Portugal: summary of participant characteristics.

	***n* (sample)**	% population^*^	**% (CI 95%)**
**Sex (*****n*** = **1,050)**			
Men	427	46.8	(41.8–51.8)
Women	623	53.2	(48.2–58.2)
**Age groups (*****n*** = **1,050)**			
18–34	82	14.6	(10.3–20.4)
35–64	602	56.7	(51.6–61.7)
65+ years	366	28.7	(24.8–32.7)
**Marital status (*****n*** = **878)**			
Married/civil union	612	67.3	(61.5–72.6)
Widow	84	7.8	(5.8–10.5)
Separated/divorced	182	24.9	(19.7–30.9)
**Occupation (*****n*** = **932)**			
Employed	463	53.7	(48.3–59.0)
Unemployed	37	4.4	(2.7–7.2)
Retired	354	32.2	(27.9–36.8)
Other situation^**^	78	9.7	(5.8–15.7)
**Chronic disease (*****n*** = 1,012)^***^			
Yes	856	82.1	(76.7–86.5)
No	156	17.9	(13.5–23.3)

^*^Weighted percentage for the population aged 18 years and over residing in Portugal in 2021 (n = 1,050).

^**^Other situation includes students, housewives, and others.

^***^Self-reported chronic conditions include asthma, allergic diseases, chronic obstructive pulmonary disease, diabetes, obesity, high blood pressure, ischemic heart disease, stroke, cancer, osteoarticular disease, depression, neurodegenerative disease, kidney disease, liver disease, spine disease, and immunity problems (including chemotherapy, transplantation, and HIV treatments).

In the 2020/2021 season, 351 individuals reported being vaccinated against seasonal influenza, corresponding to a vaccine coverage of 30.7% (CI 95%: 26.5, 35.2). IV uptake increased with age, ranging from 5.1% in those aged 18–34 years to 64.8% (CI 95%: 57.6, 71.5) among those aged 65 or more. IV coverage for individuals that reported at least one chronic condition was 35.4% (CI 95%: 30.7, 40.4) and was 49.8% for the overall target population (CI 95%: 43.6, 55.9; Table 2). Considering the target group for vaccination, significant differences were also found between individuals 65 years or more (64.8%, CI 95%: 57.6, 71.5) and individuals <65 years but with a chronic condition relevant for IV recommendation (29.4%, CI 95%: 20.9, 39.5).

**Table 2:** Health beliefs and attitudes toward influenza and COVID-19 vaccination in Portugal: IV uptake in 2020–2021 season by sex, age group, chronic condition, and target group.

	***n* (sample)**	% population^*^	**% (CI 95%)**	*p*-value^**^
Total	351	30.7	(26.5–35.2)	
**Sex (*****n*** = **1,050)**				
Men	417	29.1	(23.0–36.0)	0.502
Women	604	32.1	(26.6–38.1)	
**Age groups (*****n*** = **1,050)**				
18–34	80	5.1	(1.8–13.6)	<0.001
35–64	587	20.5	(15.7–26.2)	
65+ years	354	64.8	(57.6–71.5)	
**Chronic condition (*****n*** = **1,012)**^a^				
Yes	850	35.4	(30.7–40.4)	<0.001
No	156	9.1	(4.7–17.0)	
**Target group (*****n*** = **1,027)**^b^				
Yes	604	49.8	(43.6–55.9)	<0.001
No	423	12.8	(8.5–18.7)	
**Type of target group (*****n*** = **589)**^b^				
65+ years	354	64.8	(57.6–71.5)	<0.001
<65 years + chronic condition	235	29.4	(20.9–39.5)	

^*^Weighted percentage.

^**^Design-adjusted version of chi-square test.

^a^Self-reported chronic conditions includes asthma, allergic diseases, chronic obstructive pulmonary disease, diabetes, obesity, high blood pressure, ischemic heart disease, stroke, cancer, osteoarticular disease, depression, neurodegenerative disease, kidney disease, liver disease, spine disease, and immunity problems.

^b^Target group includes all individuals aged 65 and above and individuals aged <65 years who reported a chronic health condition.

Unvaccinated respondents (*n* = 671, 68.6%) were inquired on their reasons for not getting the IV through an open-response questions. Thematic analysis yielded 15 categories, of which two were not mapped in the HBM dimensions (Table 3), as they encompassed too vague statements about not wanting or not seeing the need for IV uptake (not providing specific reasons as to such).

**Table 3:** Health beliefs and attitudes toward influenza and COVID-19 vaccination in Portugal: motives for not taking the influenza vaccine according to the categories included in four of the Health Belief Model dimensions.

**HBM dimensions**	**Coded categories**	***n* (sample)**	% population^*^	**% (CI 95%)**	**Response units examples**
Severity		9	1.9	(0.7–5.4)	“I usually have light symptoms that go over after a few days”; “I never get really sick”
Susceptibility		170	35.9	(29.5–42.8)	
	Few flus or colds	90	15	(11–20)	“It's very rare to have a flue”; “I do not get colds in the winter”
	Age	31	9.8	(5.9–15.8)	“I think I am too young”; “not of age yet”
	Target group	27	7.1	(3.9–12.5)	“Not in the target group”; “Not a high-risk patient”
	Being healthy	15	2.7	(1.1–6.3)	“Very careful with the weather, drafts, and I am very healthy”; “I am healthy”
	Being protected	7	1.3	(0.5–3.6)	“I am working from home”; “have been using a mask”
Barriers		132	22.7	(17.7–28.5)	
	Adverse effects	28	7.3	(4.8–11)	“I got really sick the first time I took it”; “I felt worst after being vaccinated”
	Stock	27	5.8	(3.3–10)	“There was no vaccine available”; “the vaccine didn't get to the pharmacy”;
	Logistic aspects	20	3.1	(1.6–5.9)	“My doctor prescribed me, but I was never called to get the shot”; “couldn't find the time”
	Low efficacy	12	2.4	(1.0–6.1)	“The IV efficacy is usually low”; “I do not believe in the vaccine efficacy”
	Use of alternatives	11	2	(0.9–4.4)	“I take a lot of tea and take other natural medicines”; “any time I can I avoid vaccines. I defend alternative medicines”
	Fear	7	0.8	(0.3–1.9)	“I am afraid of needles and nurses”; “I am afraid of vaccines and do not like doctors”
Cues to action	Recommendation	28	6	(3.5–10)	“I was never advised to by the doctor”
Not included in HBM	No need	121	27.0	(21.2–33.8)	“Didn't think I need it”; “Do not see the need”
	Do not want it	26	4.8	(2.5–9)	“Did not want it”; “I do not want it”

^*^Weighted percentage for the population with 18 plus years residing in Portugal in 2021 that answered the open question on the reasons for not having taken the influenza vaccine (n = 496).

The different categories were included in four dimensions of the HBM as the dimension “benefits” was not addressed in the responses. The most frequent dimension was “susceptibility,” with more than a third of the non-vaccinated population belonging to a low-susceptibility category (35.9% CI 95%: 29.5, 42.8). This included having few instances of flu or cold, not belonging to the target group (according to age or absence of chronic condition), being a healthy person, or having been protected because of pandemic measures.

The second most frequent dimension was “barriers” (22.7%, CI 95%: 17.7, 28.5), which included physical barriers, such as availability in stock and logistics aspects, and internal barriers, such as consideration of adverse effects, perceived low efficacy, fear, and use of better alternatives. Both the “recommendation” and “severity” dimensions presented relatively low frequencies (6%, CI 95%: 3.5, 10.0 and 1.9%, CI 95%: 0.7, 5.4, respectively). Among the HBM categories, the most frequent reasons provided for non-uptake of the IV were having very few instances of flu or cold (15%, CI 95%: 11, 20), not being the right age (9.8%, CI 95%: 5.9, 15.8), and fear of adverse effects (7.3%, CI 95%: 4.8, 11.0). The least frequent reasons were overall fear of vaccines or doctors (1.3%, CI 95%: 0.5, 3.6.) and being protected because of pandemic measures (0.8%, CI 95%: 0.3, 1.9).

In relation to the COVID-19 vaccines, we estimated an 83.1% (*n* = 857, CI 95%: 13.6% to 20.9%) vaccine coverage with at least one dose of the vaccine by the summer of 2021 (Table 4). More than half of the respondents had already taken two doses (59.5%, CI 95%: 54.1, 64.6).

**Table 4:** Health beliefs and attitudes toward influenza and COVID-19 vaccination in Portugal: COVID-19 vaccine uptake by sex, age group, and chronic condition.

	***n* (sample)**	% population^*^	**% (CI 95%)**	*p*-value^**^
Total (one or more doses)	857	83.1	(13.6–20.9)	
One dose	180	23.6	(18.9–29.1)	
Two doses	677	59.5	(54.1–64.6)	
**Sex (*****n*** = **1,022)**				
Men	419	80.7	(74.3–85.9)	0.233
Women	603	85.2	(79.9–89.2)	
**Age groups (*****n*** = **1,022)**				
18–34	81	71.7	(55.3–83.9)	<0.001
35–64	589	80.2	(74.6–84.8)	
65+ years	352	95.2	(91.1–97.5)	
**Chronic condition (*****n*** = **1,009)**^*a*^				
Yes	854	70.5	(65.1–75.5)	<0.05
No	155	13.1	(9.1–18.6)	

^*^Weighted percentage.

^**^Design-adjusted versions of chi-square test.

^a^Self-reported chronic conditions include asthma, allergic diseases, chronic obstructive pulmonary disease, diabetes, obesity, high blood pressure, ischemic heart disease, stroke, cancer, osteoarticular disease, depression, neurodegenerative disease, kidney disease, liver disease, spine disease, and immunity problems (including chemotherapy, transplantation, and HIV treatments).

Table 5 shows the average level of agreement for each of the 13 items within the HBM model regarding vaccination against COVID-19. The dimensions “susceptibility,” “benefits,” and “cues for action” presented the higher degree of agreement (μ ≥ 4).

**Table 5:** Health beliefs and attitudes toward influenza and COVID-19 vaccination in Portugal: distribution of the population mean assessing the level of agreement for items of the Health Belief Model dimensions.

		***n* (sample)**	μ^*^	**CI 95%**
Severity	1. COVID-19 is only serious to a small part of the population	964	2.49	2.34–2.64
	2. As I am healthy, if I get COVID-19, I can take care of myself and heal	956	2.47	2.32–2.61
	3. I am afraid of getting very sick with COVID-19	993	3.66	3.51–3.81
Susceptibility	4. The most important thing to avoid COVID-19 and other diseases is to take care of oneself, our health, and immune system	1,005	4.57	4.49–4.65
	5. No matter what anyone can catch COVID-19	977	4.04	3.91–4.16
	6. I won't get COVID-19 because I am very careful with cleanliness and hygiene	987	3.09	2.96–3.23
Barriers	7. It can be difficult to schedule and go into the vaccination center for the COVID-19 shot	963	2.23	2.09–2.38
	8. No one knows the adverse effects of the vaccine on our health	946	3.63	3.48–3.77
Benefits	9. The vaccine prevents the transmission of the disease to others	944	2.97	2.82–3.12
	10. The vaccine protects people from severe complications caused by the disease	964	4.42	4.33–4.5
Cues to action	11. We catch on to the importance of vaccination for the whole population from social communication and media	983	4.24	4.13–4.35
	12. My doctor's recommendation also influences my decision to take or not take the vaccine	961	3.21	3.06–3.36
	13. I feel more willing to take the vaccine if my family and friends suggest it to me	990	2.28	2.13–2.42

^*^Weighted average.

The study participants also seemed to assess “susceptibility” inconsistently. On the one hand, there was agreement that taking care of one's health and immune system could “avoid” the transmission of the disease (Item 4; μ = 4.57, CI 95%: 4.49, 4.65), while on the other, “catching COVID-19” was perceived as an inevitability (Item 5; μ = 4.04, CI 95%: 3.91, 4.16).

The “benefits” of vaccine uptake was not so much associated with transmission of the disease as with the complications from getting sick with COVID-19. The vaccine was not perceived so much as protecting against disease transmission (Item 9; μ = 2.97, CI 95%: 2.82, 3.12) but as protecting people from severe complications caused by the disease (Item 10 μ = 4.42, CI 95%: 4.33, 4.5). The media emerged as an essential aspect to boost the vaccine uptake in the “cues for action” dimension (Item 11; μ = 4.24, 95% CI: 4.13, 4.35).

Lower levels of agreement were observed in the “barriers” and “cues for action” dimensions. The results show that scheduling the COVID-19 vaccine uptake was not perceived as difficult or a “barrier” (Item 7; μ = 2.23 CI 95%: 2.09, 2.38). The influence of the family seemed to be smaller (Item 13; μ = 2.28, CI 95%: 2.13, 2.42) when compared with the media or doctors.

In the “severity” dimension, the items suggest a high perceived disease severity of the disease, namely, because people are afraid of getting sick with COVID-19 (Item 3; μ = 3.66, CI 95%: 3.51, 3.81).

Differences in COVID-19 vaccine perception were found among the study population according to IV uptake (Table 6). While those who took the IV seemed to perceive a higher severity of COVID-19 (being afraid of getting very sick, Item 3; μ = 3.97, CI 95%: 3.77, 4.19), this subgroup also seemed to perceive lower susceptibility to the disease as they believed themselves to be careful with cleaning and hygiene (Item 6; μ = 3.34, CI 95%: 3.12, 3.57). The subgroup that took the IV tended to perceive a higher benefit of taking the COVID-19 vaccine for the severe complications caused by the disease (Item 10; μ = 3.22, CI 95%: 2.96, 3.48). Differences were also found for the “cues to action” dimension. For the individuals who reported having taken the IV shot, the importance of media, doctors' recommendations, and family and friends' suggestions was always significantly higher than for those who did not take the IV.

**Table 6:** Health beliefs and attitudes toward influenza and COVID-19 vaccination in Portugal: distribution of the population mean assessing the level of agreement for items of the Health Belief Model dimensions stratified by influenza vaccine uptake.

	**IV uptake**		
	**No** μ **(CI 95%)**	**Yes** μ **(CI 95%)**	***p*** **-value** ^**^
**Severity**			
1. COVID-19 is only serious to a small part of the population	2.43 (2.25–2.62)	2.64 (2.38–2.89)	0.2071
2. As I am healthy, if I get COVID-19, I can take care of myself and heal	2.44 (2.25–2.62)	2.53 (2.28–2.78)	0.5677
3. I am afraid of getting very sick with COVID-19	**3.53 (3.34–3.72)**	**3.97 (3.77–4.19)**	**<0.05**
**Susceptibility**			
4. The most important thing to avoid COVID-19 and other diseases is to take care of oneself, our health, and immune system	4.54 (4.43–4.64)	4.66 (4.52–4.79)	0.1609
5. No matter what anyone can catch COVID-19	4.05 (3.89–4.21)	4.03 (3.84–4.22)	0.8698
6. I won't get COVID-19 because I am very careful with cleanliness and hygiene	**2.99 (2.82–3.16)**	**3.34 (3.12–3.57)**	**<0.05**
**Barriers**			
7. It can be difficult to schedule and go into the vaccination center for the COVID-19 shot	2.21 (2.03–2.39)	2.28 (2.05–2.52)	0.6319
8. No one knows the adverse effects of the vaccine on our health	3.59 (3.41–3.77)	3.72 (3.51–3.93)	0.3453
**Benefits**			
9. The vaccine prevents the transmission of the disease to others	4.40 (4.29–4.52)	4.48 (4.30–4.65)	0.4725
10. The vaccine protects people from severe complications caused by the disease	**2.85 (2.67–3.04)**	**3.22 (2.96–3.48)**	**<0.05**
**Cues to action**			
11. We catch on to the importance of vaccination for the whole population from social communication and media	**4.15 (4.01–4.29)**	**4.44 (4.29–4.59)**	**<0.05**
12. My doctor's recommendation also influences my decision to take or not take the vaccine	**3.06 (2.87–3.24)**	**3.55 (3.29–3.80)**	**<0.05**
13. I feel more willing to take the vaccine if my family and friends suggest it to me	**2.17 (1.99–2.35)**	**2.53 (2.27–2.78)**	**<0.05**

^**^Design-adjusted version of t-test. Statistically significant results are in bold.

To understand if the differences observed for the IV uptake were related with the differences in the target groups for whom the IV is recommended, a stratified analysis was conducted (Table 7). The only difference was observed in the individuals of <65 years of age who reported a chronic condition. Within this subgroup, those who had the IV reported a higher perception of the vaccine benefits for disease transmission (Item 9; μ = 4.77, CI 95%: 4.60, 4.93), while at the same time, they showed higher doubts about the vaccine security and its adverse effects (Item 8; μ = 4.01, CI 95%: 3.64, 4.37).

**Table 7:** Distribution of the population mean assessing the level of agreement for items of the Health Belief Model dimensions stratified by two subgroups of the population (65 years and above and <65 years with a chronic disease) and influenza vaccine uptake.

	**IV uptake**					
	**65**+ **years**			<**65 years with chronic condition**		
	**No** μ **(CI 95%)**	**Yes** μ **(CI 95%)**	***p*** **-value** ^**^	**No** μ **(CI 95%)**	**Yes** μ **(CI 95%)**	***p*** **-value** ^**^
**Severity**						
1. COVID-19 is only serious to a small part of the population	2.5 (2.12–2.88)	2.78 (2.48–3.07)	0.2627	2.39 (2.03–2.74)	1.99 (1.51–2.49)	0.2106
2. As I am healthy, if I get COVID-19, I can take care of myself and heal	2.69 (2.35–3.03)	2.63 (2.34–2.93)	0.8212	2.35 (2.01–2.68)	1.87 (1.39–2.35)	0.1152
3. I am afraid of getting very sick with COVID-19	3.85 (3.47–4.22)	4.10 (3.86–4.34)	0.2528	3.84 (3.45–4.22)	3.87 (3.32–4.41)	0.9327
**Susceptibility**						
4. The most important thing to avoid COVID-19 and other diseases is to take care of oneself, our health, and immune system	4.77 (4.63–4.91)	4.77 (4.68–4.86)	0.9810	4.74 (4.63–4.85)	4.76 (4.60–4.92)	0.8352
5. No matter what anyone can catch COVID-19	4.30 (4.07–4.54)	4.12 (3.89–4.33)	0.2416	4.24 (3.97–4.52)	4.20 (3.82–4.57)	0.8363
6. I won't get COVID-19 because I am very careful with cleanliness and hygiene	3.50 (3.11–3.89)	3.65 (3.42–3.89)	0.4992	3.22 (2.87– 3.57)	2.77 (2.19–3.35)	0.1932
**Barriers**						
7. It can be difficult to schedule and go into the vaccination center for the COVID-19 shot	2.07 (1.67–2.40)	2.50 (2.21–2.78)	0.0539	2.56 (2.04–3.07)	2.35 (1.64–3.05)	0.6423
8. No one knows the adverse effects of the vaccine on our health	3.80 (3.41–4.19)	3.69 (3.45–3.94)	0.6481	**3.49 (3.17–3.81)**	**4.01 (3.64– 4.37)**	**0.0372**
**Benefits**						
9. The vaccine prevents the transmission of the disease to others	4.44 (4.26–4.63)	4.49 (4.34–4.65)	0.6783	**4.42 (4.24–4.61)**	**4.77 (4.60–4.93)**	**0.0081**
10. The vaccine protects people from severe complications caused by the disease	3.50 (3.11–3.89)	3.65 (3.39–3.91)	0.5153	3.13 (2.77–3.48)	2.56 (1.92–3.20)	0.1356
**Cues to action**						
11. We catch on to the importance of vaccination for the whole population from social communication and media	4.62 (4.43–4.82)	4.50 (4.35–4.65)	0.3320	4.30 (4.08–4.53)	4.44 (4.12–4.77)	0.4897
12. My doctor's recommendation also influences my decision to take or take not the vaccine	3.32 (2.88–3.75)	3.80 (3.53–4.06)	0.0662	2.99 (2.61–3.37)	3.07 (2.51–3.62)	0.8329
13. I feel more willing to take the vaccine if my family and friends suggest it to me	2.85 (2.44– 3.26)	2.92 (2.59–3.23)	0.8151	2.08 (1.76–2.40)	1.85 (1.46–2.23)	0.3590

^**^Design-adjusted version of t-test. Statistically significant results are in bold.

## 4 Discussion

The results show that IV uptake for the overall population was about 30.7% (CI 95%: 26.5, 35.2). Coverage was higher for the target population recommended for IV, with significant differences between older adults (64.8%, CI 95%: 57.6, 71.5), and younger individuals with a chronic condition relevant for IV recommendation (29.4%, CI 95%: 20.9, 39.5).

These results are in accordance with what has been observed in other European countries (47–51). The European Union Council recommendation and the World Health Organization recommendation established target groups for seasonal flu vaccination, including the older population, healthcare workers, people with chronic illnesses, and pregnant women (47–49, 52). However, the coverage rates vary significantly among these different target groups (49). In Europe, for the past 20 years, IV coverage in the target group has seemed to increase mainly due to the vaccination of older adults, despite not having reached the proposed 75% target (47, 49). High-risk groups of younger individuals with chronic conditions recommended for IV present overall lower rates of IV coverage (47, 49, 50). A similar trend has been observed in Portugal, with increased IV coverage in older adults but a slower and lower increase in adults with chronic conditions (53).

Vaccination campaigns may be working to increase coverage among the older population but do not seem to reach younger high-risk groups. One possible explanation is that the high-risk individuals do not recognize their conditions to be relevant for IV and do not see themselves as a target group (27).

The study results on the reasons for IV non-uptake also indicate susceptibility (35.9%; CI 95%: 29.5, 42.8) as a main factor. A rapid review including articles published between January 2012 and May 2022, which aimed to investigate the perceived barriers and attitudes to influenza vaccination, showed that, among other barriers, many participants were not opposed to the vaccine, but instead, they simply did not consider influenza to be a sufficient health threat for them (54). A meta-analysis of IV intention during the COVID-19 pandemic suggested that vaccine hesitancy is mainly due to the perceived low risk of illness combined with safety and efficacy concerns (55). Overall, influenza vaccination uptake may be determined also by perceived susceptibility, and this result can also be present either for the general population or the high-risk younger group.

The second most frequent dimension mentioned by unvaccinated individuals was “barriers,” which can be divided into external and internal barriers. Internal barriers refer to the cognitive and emotional aspects of an individual, while external barriers refer to logistic and access aspects (37). The results mainly focus on internal barriers in reference to a distrust of the vaccine (adverse effects, low efficacy, fear, and use of alternatives). When we consider these categories together, internal barriers may even be more compelling or important to IV non-uptake. It has been suggested that physical and logistic barriers only become important to those who have vaccination intention (29). This result is in accordance with other studies indicating a lack of knowledge and lack of trust of vaccines and the vaccination process as a barrier to IV (54).

Unlike the IV results, the uptake of the COVID-19 vaccine was very high in the study population, and by the end of summer 2021, it corresponded to 83.1% (CI 95%: 13.6%, 20.9%). Unlike the results for IV non-uptake, there was a high perception of the disease's possible severity and fear of the consequences. The direct benefit of the COVID-19 vaccine was, in fact, avoiding the potential more severe consequences. In a systematic review of COVID-19 vaccine hesitancy, a reverse relation between the benefits and severity dimensions of the HBM and vaccine hesitancy was observed (23). The less the perceived benefits of the vaccine and severity attributed to COVID-19, the higher the vaccine hesitancy (23). Considering the results of the present study, these seem also to be determinant factors for COVID-19 uptake in Portugal.

There was also a high perception of susceptibility to COVID-19, even though apparent contradictory results were observed. On the one hand, the population agreed that taking care of oneself health and immune system could “avoid” the transmission of the disease, while on the other, they perceived “catching COVID-19” as an inevitability. This may be due to the pandemic context and the mass communication about the disease, which was present in the daily lives of the participants during the study implementation. Awareness about COVID-19 has probably impacted the behavior and attitude of the population toward its vaccine uptake compared to the lesser media attention given to influenza vaccines and vaccination campaigns. The results from the cues to action dimension are in accordance with such a view; indeed, the media emerged as an essential aspect to boost the vaccine uptake compared to health professionals or the informal network. The role of the media, particularly non-traditional media, has been mainly discussed as a negative factor in COVID-19 uptake (5). There is a consensus, particularly on the impact of social media platforms where misinformation and disinformation seem rampant, that these platforms increase vaccine hesitancy, while other media outlets (e.g., television and newspapers) do not seem to be so frequently associated with vaccine intent (56, 57). One possible explanation for our results is the fact that media was jointly assessed as all types of outlets (including television, newspapers, and social media). Another possible explanatory factor is the importance of (23) trust in governments and scientific authorities. Studies have indicated that trust in scientific and governmental authorities is related to increased uptake (9, 23, 34); therefor, in countries with higher trust, it is to be expected that the information transmitted from these sources might also contribute to less hesitancy. Regarding vaccination, Portugal presents a higher vaccination coverage of primary scheme programme vaccines, which is indicative of such trust (52).

The pandemic context and its influence on vaccine hesitancy have been discussed in respect to either the COVID-19 vaccine, IV vaccine, or overall hesitancy (9, 10, 22, 54, 56). Some authors suggest that due to the novelty and rapid development of the COVID-19 vaccine, there was increased vaccine hesitancy even with the awareness raised as to the importance of vaccines (56). Others suggest it presented an opportunity to leverage vaccine intention to encourage uptake (9). Several studies during the pandemic have shown an increased acceptance of influenza vaccination (10, 55, 57). A rapid review of the literature on barriers to IV uptake identified a longitudinal study that reported an increase in trust in vaccines during the first and second year of the COVID-19 pandemic (2020–2021) (54).

Our results suggest that, in Portugal, IV uptake was significantly associated with health beliefs and attitudes toward the COVID-19 vaccine. Individuals who reported IV uptake seemed to perceive a higher severity of COVID-19 and a higher benefit of taking the COVID-19 vaccine for the severe complications caused by the disease. Previous influenza vaccination behavior has been shown to positively affect intention to vaccinate against COVID-19 (10). While this could also be due to the target group recommended for the IV, the stratified analysis of two high-risk subgroups highlights the association of previous IV uptake to COVID-19 vaccine perception. The only difference was observed in individuals <65 years of age with chronic conditions. Within this subgroup, those who took the IV reported a higher perception of the vaccine benefits for COVID-19 transmission.

The findings of this study reinforce the applicability of the HBM as a conceptual framework to analyze the individual beliefs and attitudes toward vaccination. Most of the open responses of the reasons for the non-uptake of the IV were categorized within the five dimensions of the model. The two dimensions not included mainly do not specify the reasons for non-uptake (they are too general to be categorized). The results of the COVID-19 attitudes and beliefs as assessed by the HBM are also aligned with the national and pandemic context. Portugal obtained a high coverage rate, and the HBM assessment in this study indicates a high perception of benefits (particularly for severe consequences) and low perceived barriers. Individuals are more likely to get vaccinated when they think they are susceptible to the disease, see a benefit from adopting the behavior, and do not perceive obstacles to getting the COVID-19 vaccine. Other studies have also supported the applicability of the HBM model to understand vaccination intention and hesitancy (23, 34, 54). This model can help understand whether attitudes and beliefs differ by vaccine and identify which dimensions have more impact on vaccination.

### 4.1 Limitations

Our study has some limitations. First are those related to the self-report, given that the data source might introduce measurement error due to memory and social desirability bias. This may, however, be minimized within the subgroup that opted for the online data collection. Second, the data collection was implemented using the CATI/CAWI mode, which requires phone and/or Internet access. Although Portugal has very high telephone and Internet coverage, during the pandemic, with the lockdowns and lay-offs and their implications on the socioeconomic status of families, some population subgroups might not have had access to these services and, as such, may not be adequately represented in our sample.

Regarding the COVID-19 vaccine, due to study timeline, our findings are limited to the primary series and might not be generalizable to booster doses. Moreover, the cross-sectional nature of the research does not allow us to determine change over time.

## 5 Conclusions

The study focuses on two different vaccines and outcomes and assesses the non-uptake of the IV and the perception of the COVID-19 vaccine using the HBM. While the methods for assessing HBM differ greatly, thus hindering comparability, the results point out important differences for the vaccination campaigns of both vaccines.

Overall, this study indicates that the population do not seem to consider influenza to be a sufficient health risk, as opposed to COVID-19, which is considered a possibly severe disease. In the case of the IV, the lack of knowledge of the disease and its consequences is important to tackle, particularly among high-risk populations. It is important not only to address the knowledge of the conditions that increase the risk of severe complications but also of the vaccines themselves; indeed, barriers related to low efficacy, fear of adverse effects, and the use of alternatives are also significant reasons for IV non-uptake.

Unlike the IV, the COVID-19 vaccine and vaccination campaigns have reached a more significant part of the population, and information on the possible severe consequences was probably more broadly disseminated. COVID-19 was mostly perceived as a very easily transmitted disease with potentially severe consequences, which could be limited by the vaccine. The role of the media in overall vaccine trust and the increased coverage rate need to be further studied. However, the present study suggests that, in the case of COVID-19, the media was an important ally, and the same could be translated into the influenza vaccination campaigns.

While the results indicate the preponderance of different HBM dimensions for the two vaccine outcomes considered, the association between IV uptake and COVID-19 perceptions highlights that an overall attitude toward vaccination in general may be an important individual determinant (22) and that self-awareness may have implications in vaccine acceptance (11).

This study indicates that influenza vaccination campaigns may need to focus more on high-risk target individuals. Secondly, in addition to specific vaccines campaigns, overall vaccine promotion may improve adherence in specific vaccines and should also be aimed at. Thirdly, the media may be a potential ally for improving vaccine coverage. It is important that persuasive messaging on vaccine effectiveness and safety, promoted by governmental and scientific institutions, is disseminated in different media outlets, particularly for the seasonal influenza vaccine.

## 6 Scope statement

The study focuses on the attitudes and beliefs toward the influenza and COVID-19 vaccinations, employing the conceptual framework of the Health Belief Model. A cross-sectional design was implemented in a representative sample of Portuguese private households included both closed- and open-ended questions to explore the perceptions and attitudes toward these two vaccines. The results show differences between the population perception of both the disease and the benefit of the vaccine and point out possible positive pandemic effects that may also be important to increase influenza vaccine uptake. The prevention of infectious disease and vaccination programs rely on individual behavior. Hence, it is important to understand and target individual determinants. The conceptual framework helps to categorize these individual determinants and compare them in relation to the different vaccinations.

## Data availability statement

The raw data supporting the conclusions of this article will be made available by the authors, without undue reservation.

## Ethics statement

The studies involving humans were approved by Portuguese Data Protection Authority (approval number 1451/2010, April 12, 2010). The studies were conducted in accordance with the local legislation and institutional requirements. The participants provided their written informed consent to participate in this study.

## Author contributions

AS: Conceptualization, Formal analysis, Methodology, Writing – original draft, Writing – review & editing. IK: Formal analysis, Methodology, Writing – review & editing. CM-D: Methodology, Project administration, Supervision, Writing – review & editing. AM: Conceptualization, Formal analysis, Methodology, Writing – review & editing.
